# Human Papillomavirus Infection and Vaccination: Awareness and Knowledge of HPV and Acceptability of HPV Vaccine among Mothers of Teenage Daughters in Weihai, Shandong, China

**DOI:** 10.1371/journal.pone.0146741

**Published:** 2016-01-14

**Authors:** Yang Yu, Minglei Xu, Jiandong Sun, Ruiying Li, Meilan Li, Jianguang Wang, Dongfeng Zhang, Aiqiang Xu

**Affiliations:** 1 Weihai Center for Disease Control and Prevention, Weihai, Shandong, PR China; 2 Medical College, Qingdao University, Qingdao, Shandong, PR China; 3 Queensland University of Technology, Brisbane, Queensland, Australia; 4 Academy of Preventive Medicine, Shandong University, Jinan, Shandong, PR China; 5 Shandong Provincial Key Laboratory of Infectious Disease Control and Prevention, Shandong Center for Disease Control and Prevention, Jinan, Shandong, PR China; Georgetown University, UNITED STATES

## Abstract

In preparation for the introduction of human papillomavirus (HPV) vaccine, we investigated awareness and knowledge of HPV/HPV vaccine and potential acceptability to HPV vaccine among mothers with a teenage daughter in Weihai, Shandong, China. A cross-sectional survey was conducted in 2013 with a sample of 1850 mothers who had a daughter (aged 9–17 years) attending primary, junior and senior high schools. In the final sample (N = 1578, response rate 85.30%), awareness of HPV was reported by 305 (19.32%) mothers. Awareness varied significantly by daughter’s age (*P<*0.01), mother’s education level (*P<*0.01), mother’s occupation (*P*<0.01), household income (*P<*0.01) and residence type (*P*<0.01). Knowledge about HPV/HPV vaccine was poor with a mean total score of 3.56 (*SD* = 2.40) out of a possible score of 13. Mothers with a higher education level reported higher levels of knowledge (*P* = 0.02). Slightly more than one-fourth (26.49%) of mothers expressed their potential acceptability of HPV vaccine for their daughters. Acceptability increased along with increased daughters’ age (*P*<0.01), household income (*P*<0.01) and knowledge level (*P*<0.01). House wives and unemployed mothers had the highest acceptability (*P*<0.01). The most common reasons for not accepting HPV vaccination were “My daughter is too young to have risk of cervical cancer (30.95%)”, “The vaccine has not been widely used, and the decision will be made after it is widely used (24.91%)”, “Worry about the safety of the vaccine (22.85%)”. Awareness and knowledge of HPV/HPV vaccines are poor and HPV vaccine acceptability is low among these Chinese mothers. These results may help inform appropriate health education programs in this population.

## Introduction

More than 270 000 women die of cervical cancer worldwide every year and over 85% of these deaths occur in low and middle income countries [1]. Cervical cancer is the third most common gynecological malignancy in China. The age-standardized incidence and mortality rate are 9.6 and 4.3 per 100,000 women respectively [2]. Human papillomavirus (HPV) infection is the main cause for the development of cervical cancer [2–6], with 70% of cervical cancers in the globe caused by HPV16 and HPV18 types [1].

The first HPV vaccine was licensed by the US Food and Drug Administration (FDA) in 2006 [7]. Currently, two HPV vaccines are licensed worldwide [8, 9] and several new multivalent HPV vaccines are under development [10]. HPV vaccination is considered to be a primary strategy for cervical cancer prevention by the World Health Organization (WHO) [1]. Australia has implemented a nationwide publicly-funded vaccination program since 2007 [7]. In the following years, the United Kingdom, Hungary and other developed countries have also introduced HPV vaccine into their national vaccination programs [11,12]. By August 2014, 58 countries had introduced HPV vaccine in their national immunization programs [13].

HPV vaccination rates in many countries have been suboptimal since HPV vaccines were licensed [14–18]. The coverage of one-dose vaccination in American adolescents aged 13–17 years increased from 9.8% in 2007 [14] to 57.3% in 2013 only [16]. Only about one-third of girls in these ages completed the immunization series (3 doses) [15]. The coverage of 3 doses in a large number of EU/EEA countries ranged from 10% to 30% in recent years [17,18].

Currently, a number of phase III clinical trials with two international and one domestic HPV vaccines are ongoing in China [2,19,20]. The prophylactic HPV vaccines are likely to be licensed in China in the near future.

According to WHO [1] and lessons learnt from other countries [7], the target population for HPV routine immunization should be primary and junior high school female students after the vaccine is licensed in China.

Before HPV vaccines are licensed, it is important to understand HPV-related awareness, knowledge and potential acceptability of HPV vaccine among both adolescent girls and significant others. The aim of this study was to explore these constructs in a highly influential population—mothers of teenage daughters to inform the forthcoming introduction of HPV vaccines in China.

## Materials and Methods

### 2.1 Study design

From November to December, 2013, we conducted two cross-sectional surveys both using self-administered anonymous questionnaires among a sample of mothers and a sample of adolescent girls, respectively. These surveys were conducted in Weihai, a city in eastern Shandong Province, PR China, with a total population of about 2.8 million. This paper reports results from the mother survey only.

The study was approved by the Ethics Review Board of the Center for Disease Control and Prevention, Shandong province. A written consent was obtained from each mother prior to the survey.

### 2.2 Study Population

This survey targeted mothers of daughters aged 9–17 years old. According to Chinese education system, the great majority of adolescents within this age range are students attending the last two grades (4–5) of primary schools (9–10 years old), and the 4 grades (1–4) of junior high schools (11–14 years old), and the 3 grades (1–3) of senior high school (15–17 years old). We used a multi-stage cluster sampling approach to determine our sample of mothers of these female students.

Firstly, all public schools with at least 2 classes in each of the aforementioned grades in the study region were identified and were grouped by school level (primary, junior and senior high) and location type (urban vs. rural / town). For each level and location type, 2 schools were approached by the research team in a convenient manner. A total of 12 schools, including 4 primary schools, 4 junior high schools and 4 senior high schools balanced by school location, agreed to participate. For each grade in these schools, 2 classes were randomly chosen and all female students’ mothers in selected classes were invited to participate in the survey.

### 2.3 Questionnaire development

The questionnaire was developed by the research team based on literature review and a pilot study. The questionnaire includes three parts: (1) demographic characteristics, such as age, education level, occupation and monthly family income et al; (2) awareness and knowledge of HPV and HPV vaccine; (3) acceptability and reasons for not accepting vaccination.

Awareness of HPV was assessed using the question “Before today, had you heard of HPV?”. Mothers who answered “yes” to this question were considered to be aware of HPV. This question has been previously used in other studies in China [2] and other countries [7, 12,17].

Knowledge of HPV and HPV vaccine was collectively assessed using 13 multiple choice questions with single correct answers. The first 8 were used to assess knowledge on HPV and the rest were used for knowledge on HPV vaccine (Table 1). Only mothers who answered “yes” to the awareness question were asked to respond to these knowledge items. Most of these questions were adopted from previous research [2, 7, 12]. Items 1, 4, 8, 13 were developed by the research team.

**Table 1:** Items assessing mothers’ knowledge of HPV / HPV vaccines.

Item number	Question	Correct answer
1	What is the HPV?	A virus
2	What is the transmission route of HPV?	Sexual transmission
3	What is the main risk factor of HPV infection?	Promiscuous sex
4	Can wives be infected with HPV in normal sexual behavior?	Yes
5	What is the main hazard of HPV infection for female?	Cause cervical cancer
6	What is the main hazard of HPV infection for male?	Cause genital warts
7	What is the main risk factor of cervical cancer?	HPV infection
8	Is the persistent HPV infection the cause of cervical cancer?	Yes
9	Can HPV vaccine effectively prevent most cervical cancer?	Yes
10	Can HPV vaccine effectively prevent genital warts of male?	Yes
11	What are the injection times of HPV vaccination?	Three times
12	What is the best time of vaccination?	Before the first sexually active
13	Is HPV vaccines effectiveness on HPV infected person?	No

The question “Are you willing to vaccinate your daughter with HPV vaccine once it is available?” was used to assess the potential acceptability to HPV vaccine. This method was also used in previous research [2,7, 12]. For mothers who answered “no” to this question were further asked to select the most likely reason for not accepting HPV vaccine using a close-ended multiple choice question with 7 specified reasons and one open choice (“Others please specify). Most of these reasons were adopted based on previous research [2,7,12].

A pilot survey with a small sample (n = 71) of mothers was conducted to test the acceptance and feasibility of the questionnaire. Based on the results from the pilot, minor wording changes were made in the final version which was then used in the main survey.

### 2.4 Data collection

The questionnaire was taken home by female students along with a written instruction. Mothers of female students were asked to fill out the questionnaire independently without consulting with any other persons or resources. Filled questionnaires were then brought back to school to the head teachers the second day.

The head teachers for selected classes agreed to collect and scan the returned questionnaire to examine 1) if there are obvious errors or unexpected answers in terms of mothers’ and daughters’ ages; and 2) if there are any missing items for other demographic questions excluding household income due to privacy concerns. In total, 17 questionnaires (8 questionnaires missed the item of education, 5 missed residence area, and 4 missed occupation) were identified by the head teachers and were returned to mothers to complete.

### 2.5. Statistical analysis

Key variables included awareness and knowledge of HPV/HPV vaccine, potential acceptability for daughters and reasons for not accepting HPV vaccination.

Correct answer to each item of HPV/HPV vaccine knowledge was assigned with 1 point; 0 point was given if the question was incorrectly answered or not answered. The possible range of the total knowledge score was 0 to 13. In regression analysis, those who did not hear of HPV and skipped these items were given a total knowledge score of zero. Finally, all respondents were divided into three groups based on their total knowledge scores: no knowledge (score = 0), low knowledge (score = 1–4) and high knowledge (score = 5–13).

The data were entered into a database developed using EpiData (EpiData Association, Odense, Denmark) independently by two persons. The SPSS 13.0 (SPSS Inc, Chicago, USA) was used to analyze the data. The Chi-square test was used for analyzing categorical data. Logistic regression analysis was used to analyze the associations between potential predictors and vaccine acceptability. Statistical significance was assessed by two-tailed tests with *α* level of 0.05.

## Results

### 3.1. Demographic characteristics

**Fig 1:**
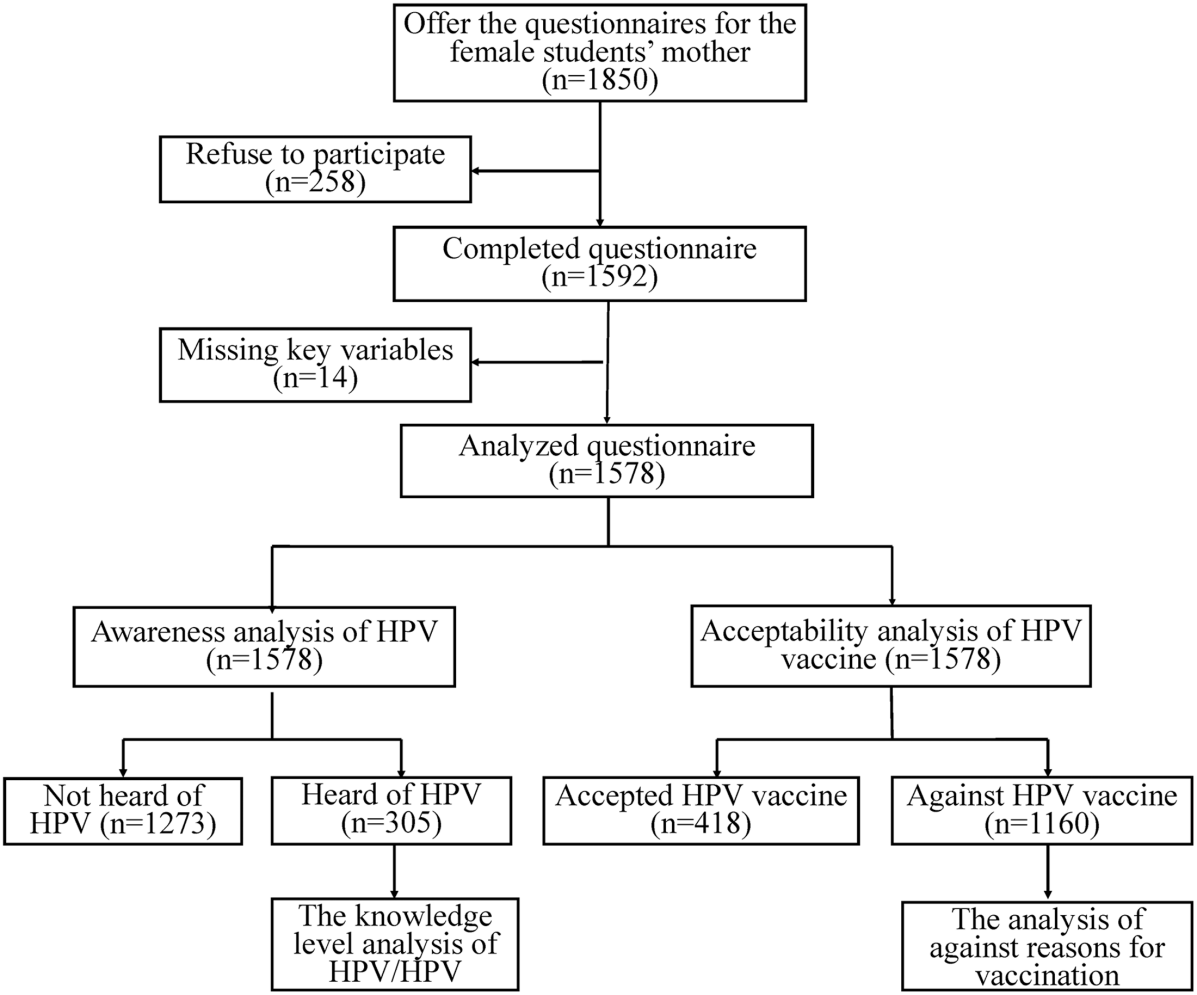
The Flow chart of study.

A total of 1850 mothers were invited to participate in this survey. With 258 mothers refusing to participate, a total of 1592 questionnaires were collected. Among these questionnaires, 14 questionnaires being excluded due to logic errors in question response (such as expressing willingness to vaccination while choosing reasons for not accepting vaccination for daughter). 1578 mothers (85.30%) were finally included in the analysis (Fig 1.).

The mean age of these mothers and daughter were 40.25±4.20 years (range: 30–56 years) and 13.03±2.44 years (range: 8–19 years), respectively. Most commonly reported education level, occupation and annual household income were junior high school, farming, and 50–100 thousand CNY (accounting for 48.80%, 21.48% and 37.52% of the total sample, respectively). Urban and rural mothers made up 38.09% and 45.94% of the sample, respectively. All demographic characteristics are presented in Table 2.

**Table 2:** The awareness rate of HPV by socio-demographic characteristics.

Socio-demographic	N(%)	Heard of HPV n(%)	Not heard of HPV n(%)	*χ*^*2*^ test
Total	1578	305(19.33)	1273(80.67)	
**Age (years)**				
<36	156(9.89)	22(14.10)	134(85.90)	χ^2^ = 4.95, *P* = 0.18
36~	766(48.54)	153(19.97)	613(80.03)	
41~	489(30.99)	103(21.06)	386(78.94)	
46~	167(10.58)	27(16.17)	140(83.83)	
**Daughter’ age (years)**				
8~	477(30.23)	87(18.24)	390(81.76)	χ^2^ = 10.88,*P*<0.01
12~	811(51.39)	142(17.51)	669(82.49)	
16~	290(18.38)	76(26.21)	214(73.79)	
**Education**				
Primary school and below	180(11.40)	31(17.22)	149(82.78)	χ^2^ = 9.48,*P*<0.01
Junior high school	770(48.80)	129(16.75)	641(83.25)	
Senior high school or above	628(39.80)	145(23.09)	483(76.91)	
**Occupation**				
Farming	339(21.48)	61(17.99)	278(82.01)	χ^2^ = 24.48,*P*<0.01
Skill worker	289(18.31)	49(16.96)	240(83.04)	
Professional	245(15.53)	75(30.61)	170(69.39)	
Business personnel	288(18.25)	51(17.71)	237(82.29)	
Housewife or unemployed	276(17.49)	43(15.58)	233(84.42)	
Other	141(8.94)	26(18.44)	115(81.56)	
Annual household income (thousand CNY)*				
<30	312(20.10)	43(13.78)	269(86.22)	χ^2^ = 12.85,*P*<0.01
30~	477(30.74)	85(17.82)	392(82.18)	
50~	592(38.14)	126(21.28)	466(78.72)	
100~	171(11.02)	44(25.73)	127(74.27)	
**Residence area**				
Urban	601(38.09)	153(25.46)	448(74.54)	χ^2^ = 23.39,*P*<0.01
Township	252(15.97)	39(15.48)	213(84.52)	
Rural	725(45.94)	113(15.59)	612(84.41)	

*26 mothers refused to report the household income.

### 3.2. Awareness and knowledge of HPV

#### 3.2.1 Awareness

Only 305 (19.33%, 95%*CI*: 17.38–21.28) mothers had heard of HPV before investigation. There was no statistically significant difference in the proportion of HPV awareness by mothers' age group (*χ*^2^ = 4.95, *P* = 0.18). Awareness was significantly more common among mothers of daughters older than 16 years (*χ*^2^ = 10.88, *P*<0.01), having an education level of senior middle school or above (*χ*^2^ = 9.48, *P*<0.01), having a professional occupation (*χ*^2^ = 24.48, *P*<0.01), with an annual household income above 100 thousand CNY (*χ*^2^ = 12.85, *P*<0.01), and residing in urban areas (*χ*^2^ = 23.39, *P*<0.01) (Table 2).

#### 3.2.2 Knowledge

Among mothers who were aware of HPV (n = 305), the mean total score was 3.56 (*SD* = 2.40) out of a possible score of 13. A total of 45 (14.75%) mothers were classified as having no knowledge related to HPV/HPV vaccine, 179 (58.69%) had low knowledge, and 81 (26.56%) had high knowledge. There were no statistically significant differences in the knowledge level by mothers’ age (*χ*^2^ = 12.37, *P* = 0.05), daughters’ age (*χ*^2^ = 5.82, *P* = 0.21), mother occupation (*χ*^2^ = 7.10, *P* = 0.72), household income (*χ*^2^ = 7.41, *P* = 0.28), and residence type (*χ*^2^ = 8.93, *P* = 0.06). Mothers with a higher level of education were more likely to be classified as having high level of HPV-related knowledge (*χ*^2^ = 12.33, *P* = 0.02) (Table 3).

**Table 3:** The level of HPV knowledge by socio-demographic characteristics (n = 305).

Socio-demographic	The level of HPV knowledge*			χ^2^ test
	n (row %) with no knowledge	n (row %) with low knowledge	n (row %) with high knowledge	
Total	45(14.75)	179(58.69)	81(26.56)	
**Age(years)**				
<36	5(22.73)	11(50.00)	6(27.27)	χ^2^ = 12.37,*P* = 0.05
36~	27(17.65)	82(53.59)	44(28.76)	
41~	11(10.67)	63(61.17)	29(28.16)	
46~	2(7.41)	23(85.18)	2(7.41)	
**Daughter’ age(years)**				
8~	13(14.94)	53(60.92)	21(24.14)	χ^2^ = 5.82,*P* = 0.21
12~	24(16.90)	74(52.11)	44(30.99)	
16~	8(10.53)	52(68.42)	16(21.05)	
**Education**				
Primary school and below	9(29.04)	18(58.06)	4(12.90)	χ^2^ = 12.33,*P* = 0.02
Junior high school	19(14.73)	82(63.57)	28(21.70)	
Senior high school or above	17(11.73)	79(54.48)	49(33.79)	
**Occupation**				
Farming	10(16.39)	39(63.92)	12(19.67)	χ^2^ = 7.10,*P* = 0.72
Skill worker	5(10.20)	32(65.31)	12(24.49)	
Professional	8(10.67)	43(57.33)	24(32.00)	
Business personnel	9(17.65)	26(50.98)	16(31.37)	
Housewife or unemployed	7(16.28)	25(58.14)	11(25.58)	
Other	6(23.08)	14(53.84)	6(23.08)	
**Annual household income (thousand CNY)**				
<30	7(16.28)	29(67.44)	7(16.28)	χ^2^ = 7.41,*P* = 0.28
30~	11(12.94)	54(63.53)	20(23.53)	
50~	23(18.25)	67(53.18)	36(28.57)	
100~	3(6.82)	27(61.36)	14(31.82)	
**Residence area**				
Urban	22(14.38)	83(54.25)	48(31.37)	χ^2^ = 8.93,*P* = 0.06
Township	2(5.13)	25(64.10)	12(30.77)	
Rural	21(18.58)	71(62.84)	21(18.58)	

*no knowledge: score = 0, low knowledge: score = 1–4; high knowledge: score = 5–13.

Among those who responded any of the knowledge questions (n = 305), nearly half or more correctly answered “What’s the main risk factor of cervical cancer?” (Item 7) (58.36%), “What is HPV?” (Item 1) (57.05%), “What’s the main hazard of HPV infection for female” (Item5) (48.85%), and “Is persistent HPV infection the cause of cervical cancer?” (Item 8) (47.54%). Poorer responses were found for the items “Can wives be infected with HPV in normal sexual behavior?” (Item 4) (17.38%), “What’s the main hazard of HPV infection for male?” (Item 6) (15.08%), “Can HPV vaccine effectively prevent genital warts for males?” (Item 10) (13.77%), “How many times does HPV vaccines need to be injected” (Item 11) (6.89%), “Are HPV vaccines effective on HPV infected person?” (Item 13) (5.25%), and “What is the best timing of HPV vaccination?” (Item 12) (4.92%) (Table 4).

**Table 4:** The awareness of knowledge among population (n = 305).

Item number	Number of mothers answered correctly	% of all mothers	95%*C*.*I*.
1	174	57.05	51.49–62.60
2	106	34.75	29.41–40.10
3	77	25.25	20.37–30.12
4	53	17.38	13.12–21.63
5	149	48.85	43.24–54.46
6	46	15.08	11.07–19.10
7	178	58.36	52.83–63.89
8	145	47.54	41.94–53.16
9	65	21.31	16.72–25.91
10	42	13.77	9.90–17.64
11	21	6.89	4.04–9.73
12	15	4.92	2.49–4.34
13	16	5.25	2.74–7.75

### 3.3 Acceptability of HPV vaccination

A total of 418 (26.49%, 95%*CI*: 24.31–28.67) mothers expressed willingness to vaccinate their daughters. Five variables were significantly associated with acceptability in Chi-square tests, including daughters’ age (*χ*^2^ = 24.33, *P*<0.01), education (*χ*^2^ = 8.79, *P*<0.01), occupation (*χ*^2^ = 32.44, *P*<0.01), household income (*χ*^2^ = 27.09, *P*<0.01), knowledge level (*χ*^2^ = 24.10, *P*<0.01). Four variables were significantly associated with acceptability in multivariate regression analysis. Increased acceptability of HPV vaccination was associated with higher daughters’ age, household income and knowledge score. The acceptability of professional and unemployed women is relatively higher (Table 5).

**Table 5:** The acceptability of HPV vaccination and logistic regression analysis.

Socio-demographic	Acceptance(n)	%	*χ*^*2*^ test	Logistic test
Total	418	26.49		
**Age(years)**				*P* = 0.42
<36	40	25.64	*χ*^*2*^ = 3.90,*P* = 0.27	1.00
36~	188	24.54		0.82(0.54–1.25)
41~	139	28.43		0.74(0.46–1.18)
46~	51	30.54		0.99(0.58–1.71)
**Daughter’ age**				*P = 0*.*00*
8~	109	22.85	*χ*^*2*^ = 24.33,*P*<0.01	1.00
12~	199	24.54		1.03(0.77–1.38)
16~	110	37.93		1.81(1.22–2.70)
**Education**				*P = 0*.*07*
Primary school and below	53	29.44	*χ*^*2*^ = 8.79,*P*<0.01	1.00
Junior middle school	178	23.12		0.65 (0.44–0.96)
Senior middle school and above	187	29.78		0.77 (0.50–1.18)
**Occupation**				*P* = 0.00
Farming	61	17.99	*χ*^*2*^ = 32.44,*P*<0.01	1.00
Skill worker	64	22.15		1.52(0.99–2.32)
Professional	78	31.84		1.71(1.09–2.68)
Business personnel	77	26.74		1.74(1.13–4.68)
Housewife or unemployed	100	36.23		2.67(1.81–3.94)
Other	38	26.95		1.75(1.07–2.87)
**Annual household income (thousand CNY)**				*P* = 0.00
<30	78	25.00	*χ*^*2*^ = 27.09,*P*<0.01	1.00
30~	104	21.80		0.85(0.60–1.21)
50~	161	27.20		1.03(0.74–1.45)
100~	72	42.11		1.90(1.23–2.93)
**Residence area**				*P = 0*.*65*
Urban	175	29.12	*χ*^*2*^ = 3.49,*P* = 0.17	1.00
Township	64	25.40		0.88(0.62–1.26)
Rural	179	24.69		1.04(0.76–1.43)
**Knowledge level**				*P* = 0.00
0	327*	24.81	χ2 = 16.96,*P*<0.01	1.00
1–4	55	30.73		1.70 (1.02–2.86)
5–13	36	44.44		2.66(1.65–4.30)

*If the participants have not heard of HPV before investigation, we consider hers HPV knowledge score was zero.

The most frequently reason for not accepting vaccination cited by mothers was “My daughter is too young to have risk of cervical cancer”(30.95%), followed by “The vaccine has not been widely used, the decision will be made after it is widely used” (24.91%), and “Worry about the safety of the vaccine”(22.84%) (Table 6).

**Table 6:** The reasons against vaccination for daughter.

Reasons against vaccination	n	%
My daughter is too young to have risk of cervical cancer.	359	30.95
The vaccine has not been widely used; the decision will be made after it is widely used.	289	24.91
Worry about the safety of the vaccine.	265	22.85
The decision is made by the children themselves.	101	8.71
Worry about the effectiveness of the vaccine.	50	4.31
The vaccine is too expensive.	44	3.79
Worry about the source of vaccine.	24	2.07
Others.	28	2.41
Total	1160	100

## Discussion

According to the recommendation by WHO [1], one targeted population of HPV vaccine should be adolescent girls after vaccines are made available in China, since girls between the ages of 13–20 years old are in the period of sexually active or pre-active, which is the optimal age for HPV vaccination [2]. Therefore, understanding awareness and knowledge of HPV/HPV vaccine, acceptability of HPV vaccine among mothers of these adolescent girls will help develop targeted interventions for cervical cancer prevention.

In this study, only 19.33% of all mothers were aware of HPV before investigation which is slightly lower than the results from another study in China (25.1%) [2] and much lower than studies from most developed countries (range: 30.0–87.7%) [7,12,17]. The lower awareness and poorer knowledge observed in our population may be associated with a number of factors. First, the demographic characteristics of this study sample are different from those studies [2,7,12,17], such as education level of other studies are higher than our study. Both this study and previous study [7] show awareness of HPV increases with increased education level. Secondly, HPV vaccine has not yet been licensed in China and HPV-related health promotion is inadequate. Most studies with higher level awareness and knowledge were carried out after HPV vaccine was introduced [7,17]. Erika et al [11] found that the educational intervention can significantly improve the Hungarian adolescents’ awareness, beliefs and attitudes of HPV. PWH Lee et al [21] conducted a study to survey the knowledge and acceptability for Chinese women in Hong Kong in 2006, before the HPV vaccine was available. Their results show that only 10% of women had heard of HPV before survey, which is even lower than our results.

We found although a number of factors are associated with the awareness of HPV, but only higher education among mothers was significantly associated with a higher level of knowledge, which is similar to another study [7]. This finding suggests that our education activities or campaigns should be focusing on mothers of lower education levels.

A large variation in the awareness was found among different knowledge items, with the correction answers ranging from 4.92% to 57.05% among mothers who heard of HPV (n = 305). In general, knowledge on HPV vaccines is poorer than that on HPV. Knowledge on HPV vaccines may be of particular importance for the introduction and implementation of vaccination programs. Such knowledge may help resist misinformation about HPV vaccines and to boost public confidence in vaccination. Future education programs should have a focus on HPV vaccine-related knowledge.

Different knowledge items may have different implications for HPV vaccination. For example, women who lack of knowledge of “Wives can be infected with HPV in normal sexual behavior” may underestimate the risk of HPV infection and refuse vaccination. In fact, the HPV infection is very common that 50% of women contract a genital HPV infection within 2 years after onset of sexual activity [22]. The infection rate is very high in China (14.2%) [23]. Mothers who don’t know “The best time for HPV vaccination” may miss the optimal timing for vaccination for their daughters. There was a trend towards earlier sexual debut and more risky sexual behaviors in China [24]. Our results further highlight the importance to develop appropriate materials for health education campaigns for mothers and other groups.

The potential acceptability (26.5%) for HPV vaccine of this study is lower than the results from another study in China (36.20%) [2] and studies from other countries (65.7–96%) [22,25]. There are several possible explanations. First of all, mothers in this study have low levels of HPV/HPV vaccine knowledge. Our analysis showed that more knowledge on HPV/HPV vaccine is significantly associated with higher acceptability which also has been demonstrated in many other studies [2,11,12,21].

Secondly, Lack of confidence in the efficacy and safety of the vaccine. The against reason of“the vaccine has not been widely using, the decision will be made after it is widely used (24.91%)” and “Worry about the safety of the vaccine (22.85%)” ranked the second and the third respectively, account for about half of the total reasons. It reflected the doubts of vaccine efficacy and safety essentially. In today’s China, more and more adverse events after vaccination were reported incorrectly, which triggers an irrational panic for vaccination safety [26].When a new vaccine was licensed for use, the people became more cautious for vaccination.

Thirdly, the cost of HPV vaccine is another obstacle for acceptance of HPV vaccine. Despite only 3.79% mothers against HPV vaccination due to the cost of HPV vaccine in this study, but the result of this study shows the higher household income, the higher acceptability of HPV vaccination. A study [27] in Australian also found that 38% of women would not vaccinate the HPV vaccine if the need to pay the cost of vaccination ($200). Another study [2] in China shows that only 25.4% parents accepted a price of 300 RMB or above. According to the foreign experience [28], HPV vaccine might be the most expensive one if it was licensed in China. The price may be an important factor to affect the HPV vaccination rate if the HPV vaccines are approved in China.

The essence of acceptability analysis is “vaccine hesitancy”. This issue is complex and context specific, varying across time, place and vaccines [29]. In this study, in addition to the misinformation, safety and effectiveness concerns, cost described above are factors that drive hesitancy, other factors also have impact on the vaccine hesitancy. For example, the result of this study shows that the acceptability of the vaccine did not increase with the increase of the education, but also decreased, which is consistent with other studies in China [2] and other countries [30,31,32]. Parents with better education background may have more access to health information including incorrect information, and thus weigh multiple factors in their decision on HPV vaccines. But the higher education as a potential barrier or a promoter to vaccine acceptance is not same in different areas and vaccine [29,30]. In addition, Even though only 19.33% of the mother had heard of HPV,but 26.49% mothers expressed willingness to vaccinate their daughters. This means that about 7% women who had not heard of HPV will vaccinate their daughters; Prior vaccination experience, the trust of the previous vaccine, especially EPI vaccine is an important promoting factor for the HPV vaccination [2]. The result of acceptability of professional and unemployed women is relatively higher is maybe the result of co-effects of education and confidence of vaccine.

There is no “magic bullet” or single intervention strategy that works for all instances of vaccine hesitancy [29]. The tailored strategies to improve vaccine acceptability should be developed basing on the different area and vaccine [30]. According to the results, we think we can improve the acceptability of HPV vaccine from three aspects before or after it is approved in China. First of all, the HPV/HPV vaccine knowledge level of the target parents must be improved; secondly, people’s concerns about safety and effectiveness should be reduced. Study have confirmed that education is the most effective way to improve the knowledge level, ease concerns about safety and effectiveness, improve the acceptability of HPV vaccine of the people[11,12]. Education via EPI program and school are the best way for the mother and adolescents respectively [2,11]. In addition, the education via public media (such as TV, newspaper, internet) cannot be ignored. Furthermore, the contents of the education are very important for the successful implementation of immunization. The contents should be correct and include the key knowledge that may affect the vaccination, such as the best vaccination time, the safety and effectiveness of vaccine etc. Thirdly, a reasonable pricing of HPV vaccine and diverse payment mechanisms should be developed. We should develop a reasonable price according to the price of other vaccines and innovative pricing and financing mechanisms after it is approved in China, such as the cost of HPV vaccine was included medical insurance in the near future and included national immune programming in the long term.

This study is strengthened by analyzing awareness, knowledge and acceptability of HPV/HPV vaccine in a large sample of Chinese mothers comprehensively. For the first time we examined some core knowledge items of HPV/HPV vaccines that were developed based on previous international and national studies and perceived importance in the Chinese social context. This will help identify specific factors that affect acceptance at a micro level, and to develop tailored vaccination strategies.

## Limitation

There are a number of limitations in our study that must be addressed. Firstly, our study was conducted only in an economically developed city in China and was not a multi-center study. Therefore our results may not be generalized to other areas, especially underdeveloped regions of China. Secondly, there may be a potential response bias in the study, since the questionnaire was filled by mothers themselves who may tend to answer socially desirable responses. Thirdly, because only a few (n = 9) mothers reported a total knowledge score of 9 or higher, we used a wide range to define the “high-level knowledge (scored 5–13) which may have resulted in some misclassification. However, one of our key results, that is, more knowledge was significantly associated with potential acceptance, remained unchanged when we removed the 7 respondents from the analysis. Finally, mothers with a daughter attending primary and senior high schools were slightly over sampled because the classes in these schools are smaller than those in junior high schools. In this study, we found positive relationships between daughters’ age and better awareness and higher acceptability. Therefore, the overall awareness and acceptability among all mothers of girls of study ages may have been overestimated.

## Conclusion

The awareness and knowledge of HPV/HPV vaccine and acceptability of HPV vaccine are poor in Shandong province of China, which may be a major obstacle for the potential introduction of HPV vaccination. The acceptability of HPV vaccination is associated with knowledge. Therefore it is critical to conduct effective education programs to raise awareness and knowledge of HPV/HPV vaccine, and in turn to boost public confidence regarding vaccine safety and effectiveness to improve the HPV vaccine acceptability.

## Supporting Information

S1 Dataset Relevant data underlying the findings described in manuscript. (XLS)
